# Normative Data for the 12 Item WHO Disability Assessment Schedule 2.0

**DOI:** 10.1371/journal.pone.0008343

**Published:** 2009-12-17

**Authors:** Gavin Andrews, Alice Kemp, Matthew Sunderland, Michael Von Korff, Tevik Bedirhan Ustun

**Affiliations:** 1 Clinical Research Unit for Anxiety and Depression, School of Psychiatry, UNSW at St Vincent's Hospital, Sydney, Australia; 2 Group Health Center for Health Studies, Group Health Cooperative, Seattle, Washington, United States of America; 3 Classifications, Terminology and Standards, World Health Organization, Geneva, Switzerland; Mount Sinai School of Medicine, United States of America

## Abstract

**Background:**

The World Health Organization Disability Assessment Schedule (WHODAS 2.0) measures disability due to health conditions including diseases, illnesses, injuries, mental or emotional problems, and problems with alcohol or drugs.

**Method:**

The 12 Item WHODAS 2.0 was used in the second Australian Survey of Mental Health and Well-being. We report the overall factor structure and the distribution of scores and normative data (means and SDs) for people with any physical disorder, any mental disorder and for people with neither.

**Findings:**

A single second order factor justifies the use of the scale as a measure of global disability. People with mental disorders had high scores (mean 6.3, SD 7.1), people with physical disorders had lower scores (mean 4.3, SD 6.1). People with no disorder covered by the survey had low scores (mean 1.4, SD 3.6).

**Interpretation:**

The provision of normative data from a population sample of adults will facilitate use of the WHODAS 2.0 12 item scale in clinical and epidemiological research.

## Introduction

This report provides normative data from a population survey using the 12 item version of the WHODAS 2.0 [1] with a simple sum scoring method. The WHODAS 2.0 is a self-report questionnaire that assesses activity limitations and participation restrictions (ie. disability) in the prior month. The WHODAS 2.0 was developed to assess six different adult life tasks: 1) Understanding and communication; 2) Self-care; 3) Mobility (getting around); 4) Interpersonal relationships (getting along with others); 5) Work and household roles (life activities); and 6) Community and civic roles (participation). There are 36 and 12 items versions of the WHODAS 2.0 that can be completed by the patient, by their clinician, or by an informant.

The WHODAS 2.0 was developed to assess difficulties due to health conditions including diseases, illnesses, or injuries, mental or emotional problems, and problems with alcohol or drugs. The WHODAS 2.0 does not attempt to determine whether disability is due to physical or psychological disorders. The WHODAS 2.0 (like other generic disability measures that are not disorder-specific) has generally found the disability associated with mental disorders to be equal to or greater than that associated with physical disorders, depending on the specific mental and physical disorders being compared [2].

The internal consistency and test-retest reliability of the overall WHODAS 2.0 scores are high, suggesting potential utility in assessment of individual patients as well as assessing group differences. The concurrent validity of the WHODAS 2.0 in comparison to other disability measures has been established in a wide range of patient populations, in general population samples, and across different countries and languages of administration. Evaluation of responsiveness to change indicates that the WHODAS 2.0 performs across diverse chronic conditions as well as, and often better than, SF-36 sub-scales assessing disability [3].

Generic self-report measures of health-related disability are often found to have less responsiveness to change than disorder-specific measures. With these caveats in mind, the WHODAS 2.0 was found to be as responsive to change as disorder-specific functional measures in several studies [3]. Furthermore, a growing body of research has evaluated the agreement of self-report measures of disability with objective measures of disability, finding good agreement [4]–[6].

A psychometric evaluation of a preliminary version of the 12 item WHODAS 2.0 confirmed that each of the six life tasks was strongly correlated with an underlying *Global Disability* latent variable [1]. We replicate this analysis by fitting confirmatory factor models to the data from the current 12 item version of the WHODAS 2.0.

**Figure 1:**
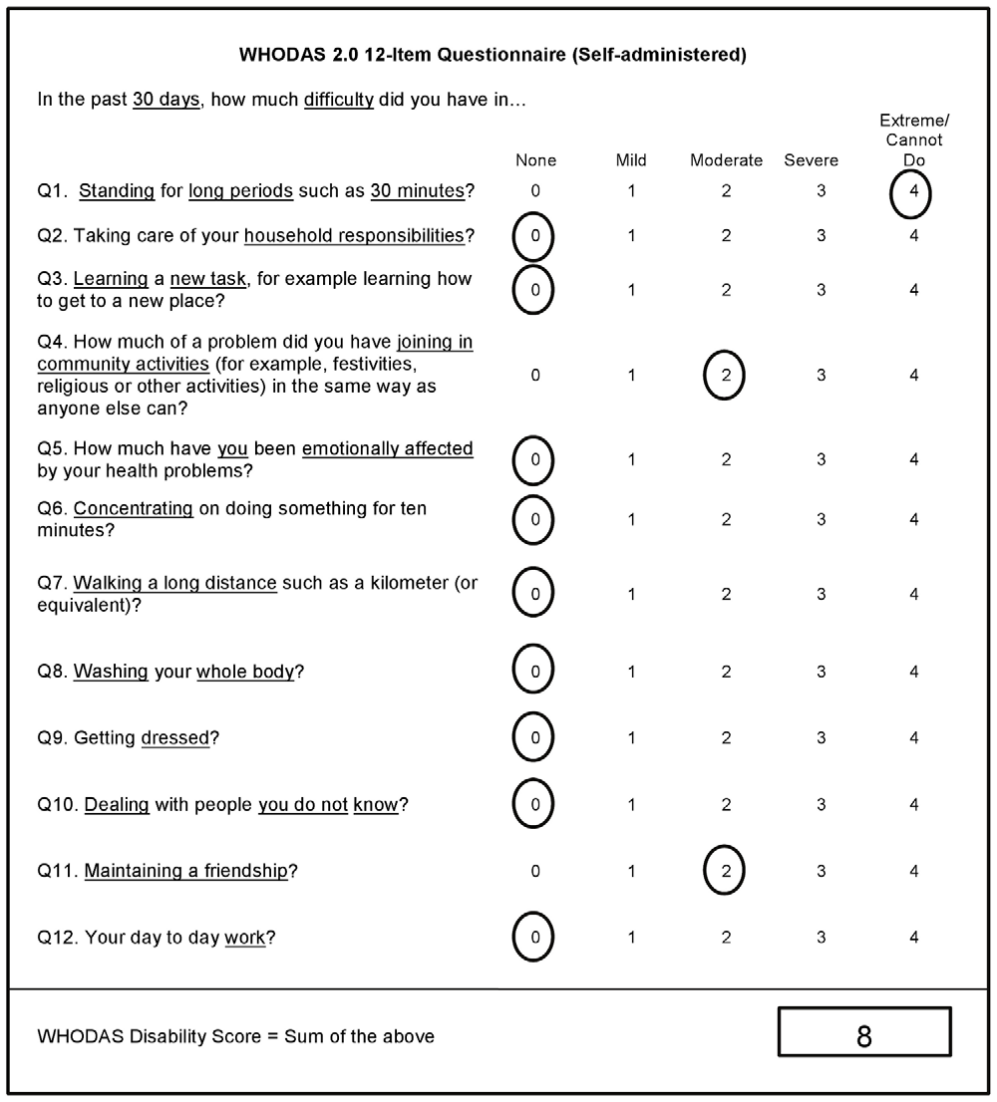
WHODAS-2.0 12 item self-administered questionnaire scoring example. Text downloaded from7 www.who.int/icidh/whodas/instrument_download.html (27/5/09)

The WHODAS 2.0 originally had a weighted scoring method based on Item Response Theory (IRT). This scoring method involved recoding specific items before converting the total score into a percentage. It was hypothesized that a simple sum method of scoring the WHODAS 2.0 12 item may improve its ease of use and facilitate hand scoring, particularly during clinical administration. We used data from two clinical samples of patients admitted to an online and face-to-face treatment program for the anxiety and depressive disorders as well as a representative sample of elderly residents, all three groups from Australia. The data was scored using both scoring methods and compared to each other using Pearson's correlation and the Bland-Altman method for assessing agreement between two measures [7]. Correlations between the two methods were consistently high in all three samples (>.98). Furthermore, we found considerable agreement between the two scoring methods with only minor variations observed in final scores. We concluded that scoring the WHODAS 2.0 12 item using a simple sum scoring method produces similar scores and does not substantially alter the interpretation. The sum score for global disability therefore ranges from 0 (no disability) to 48 (complete disability). The 12 item WHODAS 2.0 and the scoring method is displayed in Figure 1. This paper provides age and sex-specific norms for the WHODAS 2.0 12 item version based on a population sample of Australian adults.

## Methods

The WHODAS 2.0 was included in the 2007 Australian National Survey of Mental Health and Well-being conducted by trained interviewers from the Australian Bureau of Statistics. A multistage stratified sample of households was contacted, and the specific person to be interviewed was identified. The survey oversampled younger people (16–24 years) and older people (65–85 years) to improve the reliability of estimates for these groups. This sampling process yielded 8,841 fully-responding households [8]. Mental disorders (affective, anxiety and substance use disorders) present in the past twelve months were identified from responses to the World Mental Health Composite International Diagnostic Interview (CIDI). Seven classes of chronic physical conditions (any cancer, diabetes, cardiovascular disease, digestive disorders, musculoskeletal conditions, respiratory problems, or hearing or vision impairment) as assessed by the World Mental Health CIDI to be present in the past twelve months were identified.

The factor structure was explored to confirm the importance of the WHODAS 2.0 as a general measure of activity limitation. First, we did an exploratory Principal Components Analysis (PCA) with oblique rotation (direct oblimin) of polychoric correlations on a random 50% of the sample. Kaiser-Mayer-Olkin's (KMO) measure of sampling adequacy was computed to assess the suitability of the data for factor analysis. Values above 0.6 provide sufficient evidence for the factorability of the correlation matrix [9]. Additionally, Bartlett's test of sphericity was calculated to test whether the correlation matrix was an identity matrix (i.e. no relationship between the items). Second, data from the remaining 50% were used to conduct Confirmatory Factor Analyses (CFA) of polychoric correlations to compare the model identified in the exploratory analysis with a theoretically derived model that posited both a general disability factor and factors related to the six domains of information in the questionnaire. The models were fitted using robust diagonally weighted least squares method of estimation recommended for the analysis of ordinal data. Good model fit is evidenced by a combination of the Tucker-Lewis fit index (TFI>0.90), the comparative fit index (CFI>0.90), the standardized root mean-square residual (SRMR≤0.08), and the root means square error of approximation (RMSEA≤0.08). Finally, to compare nested models the Akaike Information Criterion (AIC) was generated. Generally, the model with the smallest AIC value has the better fit. The statistical models were fitted using the Statistical Package for Social Sciences (SPSS) version 17.0 and LISREL version 8.80.

The distributions of the disability scores on the WHODAS 2.0 were examined and differences between means were assessed by t-tests and two-way ANOVAs, which have been found to be suitable for highly skewed data when the sample size is large [10]. The data were weighted to the Australian general population and jack-knife replicate weights were used for statistical estimation to take into account the sampling error arising from the complex survey design. All analyses were conducted using the SUDAAN statistical software package version 10.

## Results

**Figure 2:**
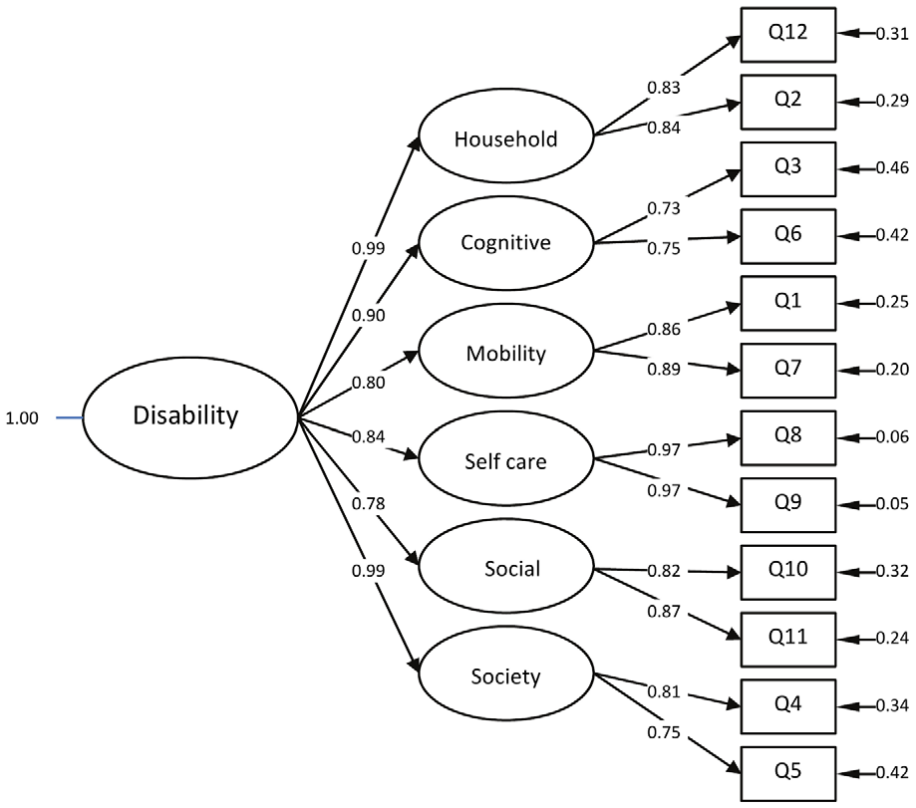
Path Diagram of a second-order factor model for the WHODAS 2.0 12 item.

8841 adults aged 16–85 participated, representing a sixty percent response rate, but 17 participants had missing data on one or more of the WHODAS 2.0 items and were excluded from all analyses. KMO's measure of sampling adequacy was 0.92 and Barlett's test of sphericity was significant (χ^2^ = 45003, df = 66, p<0.001) indicating that factor analysis was suitable for this data. Principal Components Analysis generated two factors with eigenvalues above 1.0 (7.21, 1.32), but inspection of the screeplot provides sufficient evidence for one strong factor solution. All twelve items loaded >0.60 on the one factor. A set of two models were estimated and compared using Confirmatory Factor Analysis; 1) a one factor solution with all items loading on one disability factor as indicated in the PCA; 2) A theoretical hierarchical solution that included a single second-order factor representing disability, and six first-order factors that represent the six domains of disability. The one-factor solution did not fit the data well (TFI = 0.97, CFI = 0.98, SRMR = 0.11, RMSEA = 0.09, AIC = 2029). The introduction of a second-order one factor solution with six first-order factors, displayed in Figure 2, provided improved model fit (TFI = 0.99, CFI = 1.00, SRMR = 0.07, RMSEA = 0.04, AIC = 546) and was chosen as the best fitting model, thereby justifying the use of general disability norms rather than individual subscale norms.

The WHODAS 2.0 distribution of mean scores by age group and sex is displayed in Table 1. The mean for the whole population was 3.1 (SD = 5.3, Skew = 3.1, range 0–48) and the distribution of percentile scores by age group are displayed in Table 2. Forty five percent of the population scored 0, that is, they reported no difficulty in any activity, 34% of the population scored 1–4, 12% scored 5–9, and nearly 10% scored between 10 and 48. The scores increased with age after controlling for gender (Wald F = 15.23, p<0.001). Women scored slightly higher than men overall (t = 2.92, p = 0.005), but the difference was not significant after controlling for age (Wald F = 0.04, n.s). There was no significant interaction between age and gender (Wald F = 1.00, n.s).

**Table 1:** WHODAS 2.0 12 item simple sum mean scores and standard deviations (SD) for the total population by age group.

	Women		Men		Total	
Age Group	**N**	**Mean (SD)**	**N**	**Mean (SD)**	**N**	**Mean (SD)**
16–24	790	2.7 (4.7)	680	1.9 (3.7)	1470	2.3 (4.2)
25–34	774	2.5 (4.3)	515	2.5 (5.8)	1289	2.5 (5.1)
35–44	882	2.8 (4.8)	756	2.8 (5.0)	1638	2.8 (4.9)
45–54	697	3.8 (6.1)	565	2.6 (4.7)	1262	3.2 (5.5)
55–64	667	3.5 (5.6)	602	3.3 (5.8)	1269	3.4 (5.7)
65–74	529	3.9 (5.7)	571	3.5 (5.4)	1100	3.7 (5.5)
75–85	467	5.7 (6.8)	329	5.6 (7.5)	796	5.7 (7.1)
Total	4806	3.3 (5.4)	4018	2.8 (5.3)	8824	3.1 (5.3)

**Table 2:** WHODAS 2.0 12 item simple sum scores at the 50^th^ through 95^th^ percentiles for the total population.

Total Population					
Age Group	50th	75th	85th	90th	95th
16–24	1	3	5	7	11
25–34	0	3	5	7	11
35–44	1	3	7	9	14
45–54	1	4	7	10	15
55–64	1	4	7	10	15
65–74	1	5	8	12	16
75–85	3	8	12	15	22
Total	1	4	7	9	14

### Mental Disorders

Seventeen percent of respondents (n = 1540) reported symptoms that matched diagnostic criteria for at least one common mental disorder (mood, anxiety or substance use disorder) present in the past 12 months. The mean WHODAS 2.0 score for people with a common mental disorder was 6·3 (SD = 7.1, range 0–48), twice that of the population as a whole. Only 19% reported no difficulty in any activity, half that of the population as a whole. Distributions of mean scores for people with any, one, or more than one concurrent mental disorder, are presented in Table 3, and distributions of percentiles are presented in Table 4.

**Table 3:** WHODAS 2.0 12 item simple sum mean scores and standard deviations (SD), by age group for people with any 12 month mental disorder, and separately for people with 1 and more than 1, 12-month mental disorder.

	Any Mental Disorder		1 Mental Disorder		>1 Mental Disorder	
Age Group	**N**	**Mean (SD)**	**N**	**Mean (SD)**	**N**	**Mean (SD)**
16–24	356	4.4 (5.9)	210	3.0 (5.6)	146	6.3 (5.8)
25–34	290	5.2 (6.1)	156	3.5 (4.2)	134	7.2 (7.2)
35–44	337	6.4 (7.0)	182	3.7 (4.7)	155	9.5 (7.9)
45–54	252	7.3 (7.6)	125	5.0 (6.4)	127	10.0 (8.1)
55–64	185	8.9 (8.6)	96	7.6 (8.7)	89	10.4 (8.3)
65–74	79	8.3 (7.5)	52	7.1 (6.2)	27	10.9 (9.0)
75–85	41	10.3 (8.1)	28	9.0 (6.3)	13	13.3 (10.6)
Total	1540	6.3 (7.1)	849	4.4 (6.0)	691	8.7 (7.7)

**Table 4:** WHODAS 2.0 12 item simple sum scores at the 50^th^ through 95^th^ percentiles for people with any 12 month mental disorder, and separately for people with 1 and more than 1 12-month mental disorder.

**Any Mental Disorder**					
Age Group	50th	75th	85th	90th	95th
16–24	3	6	9	12	15
25–34	3	8	11	13	17
35–44	4	10	15	17	21
45–54	5	11	16	19	23
55–64	7	13	17	21	25
65–74	7	13	17	18	23
75–85	9	13	17	20	30
Total	4	9	13	16	21
**1 Mental Disorder**					
Age Group	50th	75th	85th	90th	95th
16–24	1	4	5	7	10
25–34	2	5	8	10	13
35–44	2	5	8	9	14
45–54	2	7	10	14	19
55–64	5	11	16	19	25
65–74	6	12	14	17	17
75–85	9	13	15	16	21
Total	2	6	9	12	16
**>1 Mental Disorder**					
Age Group	50th	75th	85th	90th	95th
16–24	5	11	14	15	16
25–34	6	10	13	17	23
35–44	7	16	18	20	26
45–54	8	15	18	23	26
55–64	8	14	18	24	25
65–74	8	18	23	24	29
75–85	9	17	30	30	38
Total	7	13	16	19	24

Many of the people with common mental disorders had concurrent physical disorders and the WHODAS 2.0 score would reflect this comorbidity. We identified the sub group (n = 609) who had a mental but no physical disorder. Their disability score was less (M = 4.2, SD = 5.2) but still significantly higher than that of the remainder of the total population (M = 3.0, SD = 5.3; t = 3.90, p<0.001).

### Physical Disorders

Fifty four percent of respondents (n = 4750) reported at least one chronic physical condition. The mean for people with a chronic physical condition was 4.3 (SD = 6.1, range 0–48), less than those for people with mental disorders but greater than the total population. One third (33%) reported no difficulty in any activity. Distributions of mean scores for people with any, or one or more than one chronic physical condition, are presented in Table 5, and distributions of percentiles are presented in Table 6.We identified the sub group (n = 3819) who had a physical but no mental disorder. Again, their disability score was less (M = 3.4, SD = 5.2), but still significantly higher than that of the remainder of the total population (M = 2.9, SD = 5.4; t = 3.19, p = 0.002).

**Table 5:** WHODAS 2.0 12 item simple sum mean scores and standard deviations (SD), by age group for people with any chronic physical condition, and separately for people with 1 and more than 1, chronic physical condition.

	Any Physical Condition		1 Physical Condition		>1 Physical Condition	
Age Group	**N**	**Mean (SD)**	**N**	**Mean (SD)**	**N**	**Mean (SD)**
16–24	397	3.7 (5.6)	294	2.9 (4.5)	103	5.9 (7.6)
25–34	475	3.4 (5.3)	331	2.7 (3.9)	144	5.2 (7.6)
35–44	711	4.1 (6.0)	448	2.9 (4.7)	263	5.9 (7.3)
45–54	727	4.5 (6.3)	397	3.3 (5.7)	330	5.9 (6.6)
55–64	881	4.2 (6.0)	392	2.5 (4.5)	489	5.6 (6.7)
65–74	885	4.1 (5.8)	376	2.7 (4.6)	509	5.2 (6.3)
75–85	674	6.2 (7.4)	207	4.1 (6.4)	467	7.2 (7.6)
Total	4750	4.3 (6.1)	2445	3.0 (4.9)	2305	5.8 (7.0)

**Table 6:** WHODAS 2.0 12 item simple sum scores at the 50^th^ through 95^th^ percentiles for people with any chronic physical condition, and separately for people with 1 and more than 1 chronic physical condition.

**Any Physical Condition**					
Age Group	50th	75th	85th	90th	95th
16–24	2	5	8	11	15
25–34	1	4	8	9	14
35–44	1	5	10	12	18
45–54	2	6	10	13	18
55–64	2	6	10	12	16
65–74	2	6	9	12	17
75–85	4	9	13	17	23
Total	2	6	10	12	17
**1 Physical Condition**					
Age Group	50th	75th	85th	90th	95th
16–24	1	4	6	8	15
25–34	1	4	7	9	11
35–44	1	4	7	9	11
45–54	1	4	7	9	16
55–64	1	3	5	7	12
65–74	1	4	6	8	12
75–85	2	5	9	11	21
Total	1	4	6	9	13
**>1 Physical Condition**					
Age Group	50th	75th	85th	90th	95th
16–24	3	8	12	14	19
25–34	2	7	12	14	23
35–44	2	9	15	18	21
45–54	4	9	12	15	19
55–64	3	9	11	13	19
65–74	3	7	12	14	18
75–85	5	10	14	17	23
Total	3	9	12	15	20

Thirty nine per cent of respondents (n = 3465) reported none of the physical or mental disorders covered by the survey and they are the ‘well group’, although some may have suffered from rarer diseases not covered by the survey. The mean for ‘well people’ was 1.4 (SD = 3.6, range 0–48), less than those for people with mental or physical disorders. The distribution was skewed (skew = 7.0), and 63% reported no difficulty in any activity.

## Discussion

The factor structure confirmed that each of the six life tasks was strongly correlated with an underlying *Global Disability* latent variable. The original WHODAS 2.0 IRT based scoring method used weighted scores that could result in an additional level of complexity when scoring by hand in a clinical situation. We have shown that a simple sum scorer, as displayed in Figure 1 and reported in this article, gives scores that have comparable psychometric properties to that of the original scorer. The raw score can be converted into a percentile score, to yield an approximate comparison with older published data by simply doubling the raw score.

This paper provides normative data for the 12 item version of the WHODAS 2.0. There is no agreed upon cut-point for identifying persons with significant disability, but persons scoring 10–48 are in the top 10% of the population distribution of WHODAS 2.0 scores and are likely to have clinically significant disability. Forty-five percent score 0 with no reported activity limitations, but whether the 34% scoring 1–4 should be regarded as showing mild, or the 12% scoring 5–9 as showing moderate disability depends to some extent on the distribution of the scores among the items in the questionnaire.

There is evidence that there is cross-national variation in norms for disability measures [3]. Normative data should therefore be developed for different countries for the 12 item version of the WHODAS 2.0 to provide fine grained country-specific normative data.

In conclusion, the 12 item version of the WHODAS 2.0 provides a brief, reliable and valid measure of global disability for use in epidemiological and health services research. This paper provides population normative data that may be used to determine where adults fall in the WHODAS 2.0 distribution by age, sex and morbidity status.
